# Vitamin D levels in an Australian and New Zealand cohort and the association with pregnancy outcome

**DOI:** 10.1186/s12884-018-1887-x

**Published:** 2018-06-20

**Authors:** Rebecca L. Wilson, Alison J. Leviton, Shalem Y. Leemaqz, Paul H. Anderson, Jessica A. Grieger, Luke E. Grzeskowiak, Petra E. Verburg, Lesley McCowan, Gustaaf A. Dekker, Tina Bianco-Miotto, Claire T. Roberts

**Affiliations:** 1 Robinson Research Institute, University of Adelaide, Adelaide, South Australia Australia; 2 Adelaide Medical School, University of Adelaide, Adelaide, South Australia Australia; 3 School of Pharmacy and Medical Sciences, Division of Health Sciences, University of South Australia, Adelaide, South Australia Australia; 4 University Medical Center Groningen, University of Groningen, Groningen, The Netherlands; 5 Department of Obstetrics and Gynaecology, University of Auckland, Auckland, New Zealand; 6 Women’s and Children’s Division, Lyell McEwin Hospital, Elizabeth Vale, South Australia Australia; 7 Waite Research Institute & School of Agriculture, Food and Wine, University of Adelaide, Adelaide, South Australia Australia

**Keywords:** Vitamin D, Pregnancy, Pregnancy outcome, Gestational diabetes mellitus, Fetal sex

## Abstract

**Background:**

Pregnant women are at increased susceptibility to vitamin D deficiency. Hence, there is continuing interest in determining how vitamin D influences pregnancy health. We aimed to compare vitamin D status in two distinct populations of pregnant women in Australia and New Zealand and to investigate the relationship between vitamin D status and pregnancy outcome. This included evaluating possible effect measure modifications according to fetal sex.

**Methods:**

Serum 25-hydroxy vitamin D (25(OH)D) was measured at 15 ± 1 weeks’ gestation in 2800 women from Adelaide and Auckland who participated in the multi-centre, prospective cohort SCreening fOr Pregnancy Endpoints (SCOPE) study.

**Results:**

Mean serum 25(OH)D in all women was 68.1 ± 27.1 nmol/L and 28% (*n* = 772) were considered vitamin D deficient (< 50 nmol/L). Serum 25(OH)D was lower in the women recruited in Adelaide when compared to the women recruited in Auckland and remained lower after adjusting for covariates including maternal body mass index and socioeconomic index (Adelaide: 58.4 ± 50.3 vs. Auckland: 70.2 ± 54.5 nmol/L, *P* < 0.001). A 53% decreased risk for gestational diabetes mellitus (GDM) was observed with high (> 81 nmol/L) “standardised” vitamin D status when compared to moderate-high (63–81 nmol/L, aRR, 0.47; 95% CI: 0.23, 0.96). Marginal sex-specific differences occurred between vitamin D status and GDM: women carrying a female fetus had a 56% decreased risk for GDM in those with low-moderate levels of standardised vitamin D (44–63 nmol/L) compared to moderate-high levels (aRR: 0.44; 95% CI: 0.20, 0.97), whilst in women carrying a male fetus, a 55% decreased risk of GDM was found with high standardised vitamin D when compared to moderately-high vitamin D, but this was not statistically significant (aRR: 0.45; 95% CI: 0.15, 1.38).

**Conclusions:**

High serum 25(OH)D at 15 ± 1 weeks’ gestation was shown to be protective against the development of GDM. A possible association between fetal sex, vitamin D status and GDM provides further questions and encourages continual research and discussion into the role of vitamin D in pregnancy, particularly in vitamin D replete populations.

**Electronic supplementary material:**

The online version of this article (10.1186/s12884-018-1887-x) contains supplementary material, which is available to authorized users.

## Background

With an increasing prevalence of vitamin D deficiency and insufficiency reported both in Australia and New Zealand, as well as worldwide [1], there is continuing interest in determining how vitamin D deficiency may influence health in pregnancy. Evidence suggests that vitamin D deficiency is associated with a number of pregnancy complications including preeclampsia (PE), gestational diabetes mellitus (GDM) and spontaneous preterm birth (sPTB) [2–4]. However, inconsistencies between studies reflect uncertainty about the true effect of vitamin D deficiency on pregnancy outcome [5, 6]. This may be explained, in part, by inadequate control of related risk factors and confounders in statistical analyses, variations between assays that measure vitamin D and significant heterogeneity between studied populations [6].

Vitamin D status is determined by measuring circulating serum levels of 25-hydroxy vitamin D_2 + 3_ (25(OH)D). In Australia and New Zealand, the deficiency cut-offs are based on the role of vitamin D in bone health where serum 25(OH)D ≥ 50 nmol/L at the end of winter is required for optimal musculoskeletal health [1]. Furthermore, it has been established that serum 25(OH)D ≥ 50 nmol/L is recommended during pregnancy and lactation [7]. The incidence of vitamin D deficiency (˂50 nmol/L) is frequent among pregnant women even in areas such as Australia and the North Island of New Zealand where sunlight exposure is high. Studies focused on high-risk populations, for example, veiled, dark-skinned or obese women in Australia and New Zealand, report between 50 and 94% of women to be vitamin D deficient [8–10]. Reports from lower-risk groups have indicated that vitamin D deficiency occurs in 25–55% of pregnant women [11–13].

There are numerous studies which have shown that vitamin D deficiency is associated with adverse pregnancy outcomes (Most recent: [14–17]), particularly in populations that reside at higher latitudes. However, studies in women from Australia and New Zealand are less consistent. Previous studies on pregnant Australian and New Zealand women have reported that while circulating 25(OH)D was significantly lower in women with PE, sPTB, GDM and those who delivered a small-for gestational age (SGA) infant, no association between vitamin D deficiency and these pregnancy complications was found after adjusting for covariates [18–20]. Differences in ethnicity [21], solar exposure and geographical location, as well as genetics [22] and supplementation [23–25], are known to affect 25(OH)D status and therefore influence study outcomes. Furthermore, the gestation at which vitamin D was measured is also important. In the case of preterm delivery, vitamin D status measured closer to the delivery date was more significantly associated with preterm delivery than earlier measures [26]. However, measuring circulating 25(OH)D in early pregnancy is common and potentially clinically useful as this is prior to when many of the pregnancy complications that affect late gestation manifest.

Using a robust, validated chemiluminescent-based assay to measure serum 25(OH)D [27], we aimed to investigate the differences between vitamin D status in early pregnancy in two distinct populations of nulliparous women from Australia and New Zealand. We also aimed to examine the relationship between serum 25(OH)D at 15 ± 1 weeks’ gestation and the risk of an adverse pregnancy outcome and included determining the effect modification of fetal sex on the association between maternal vitamin D status and pregnancy outcome.

## Methods

### Study population

This study utilised data collected from the multi-centre, prospective cohort Screening for Pregnancy Endpoints (SCOPE) study [28]. Nulliparous women carrying a singleton pregnancy were recruited between November 2004 and September 2008 in Adelaide (Australia) and Auckland (New Zealand). Ethics approval was obtained from Central Northern Adelaide Health Service Ethics of Human Research Committee on 2 September 2005 (ethics number REC 1714/5/2008) and Northern Region Ethics Committee, in Auckland on 23 April 2003 (ethics number AKX/02/00/364). All participants provided written informed consent. At 15 ± 1 weeks’ gestation, women were interviewed by a research midwife and asked questions on maternal demographics and lifestyle and had physical measurements taken, including height and weight. Records included ethnicity, age, body mass index (BMI), socioeconomic index (SEI) and multivitamin use [29]. BMI was calculated as weight (kg)/height^2^ (m^2^). Obesity was defined as BMI ≥30 kg/m^2^, overweight as BMI ≥25 and < 30 kg/m^2^, normal weight as BMI > 20 and < 25 kg/m^2^ and underweight as BMI ≤20 kg/m^2^. SEI was calculated using the New Zealand SEI of occupational status, deriving a number between 10 and 90 based on the woman’s occupation; a higher number indicates higher socioeconomic status [30]. This population of women was predominantly Caucasian and thus maternal ethnicity was categorised into 2 main groups; Caucasian and non-Caucasian. Uncomplicated pregnancies were defined as those without any pregnancy disorder who delivered an appropriate weight for gestational age infant at term (≥37 weeks gestation) [31]. Pregnancy complications studied included PE, gestational hypertension (GH), GDM, sPTB and SGA and have been previously defined [28, 29, 31, 32].

### Measurement of serum 25(OH)D

Non-fasting whole peripheral blood samples were collected into non-heparinised tubes at 15±1 weeks’ gestation. Serum was processed within 4 h of collection and stored at − 80 °C until required. Unlike the previously published data on vitamin D in the Auckland cohort [20], serum 25(OH)D was measured using the IDS-iSYS chemiluminescent-based assay (Abacus, ALS) as per the manufacturer’s instructions. Average intra- and inter-assay coefficient of variation (CV) were 5.4 and 8.8%, respectively. Both serum 25(OH)D_3_ and 25(OH)D_2_ were measured independently and combined to provide a total 25(OH)D in nmol/L [27].

### Statistical analyses

Statistical analyses were performed in R (v3.1.1) [33]. Data were checked for normality using a Shapiro-Wilks test and differences between women recruited in Adelaide compared to Auckland were tabulated and compared using a Welch’s t-test (continuous variables) or Fisher’s exact test (categorical variables).

Given that the month in which the serum was sampled heavily influences vitamin D status, “standardised” serum 25(OH)D concentrations were calculated as previously described [34] in order to normalise against seasonal variation. Briefly, vitamin D concentrations were standardized by taking the difference of 25(OH)D concentration to the average 25(OH)D concentration of the corresponding month. This 25(OH)D concentration was then added to the overall population mean 25(OH)D concentration: 68.09 nmol/L. Quartiles, based on the distribution amongst the population of women studied, in the standardised concentrations were then used to create cut-points and designated ‘low’ (< 44 nmol/L), ‘low-moderate’ (44–63 nmol/L), ‘moderate-high’ (63–81 nmol/L) and ‘high’ (> 81 nmol/L) categories of serum 25(OH)D.

Generalised linear models (Poisson with log link and robust variance estimates) were used to calculate the risk ratios for pregnancy complications by standardised serum 25(OH)D concentrations calculated among the women who had an uncomplicated pregnancy. Potential confounders for vitamin D status were assessed using linear modelling. Maternal age, BMI, SEI, alcohol consumption at 15 ± 1 weeks’ gestation (never/former vs. current), recreational walking (1–3 times/week and ≥ 4 times/week vs. never), ethnicity (Caucasian vs. non-Caucasian) and recruitment site (Adelaide vs. Auckland) were significantly associated with serum 25(OH)D and along with smoking status at 15 ± 1 weeks’ gestation (never/former vs. current) included as main effects within the generalised linear models. These analyses were also repeated but using the current definitions of vitamin D deficiency: < 25, 25–50, 50–75 and > 75 nmol/L in the non-standardised data [7]. We also stratified based on fetal sex to evaluate possible effect measure modifications according to whether the mother was carry a male or female fetus.

## Results

### Population characteristics

Of 3229 women recruited as part of SCOPE at the Adelaide and Auckland centres, serum samples at 15 ± 1 weeks’ gestation to measure 25(OH)D were available for 2800 (87%) women of whom, 1156 (41%) were recruited in Adelaide and 1644 (59%) were recruited in Auckland. Maternal characteristics are shown in Table 1. Compared to women who were recruited at the Adelaide site, women recruited in Auckland were older, had a lower BMI, less likely to smoke or drink alcohol during pregnancy and more likely to eat fruit and undertake recreational walks. Mean ± SD SEI of the Auckland women was also higher compared to that in women recruited in Adelaide (Auckland: 48.0 ± 14.8 vs. Adelaide: 27.7 ± 10.5, *P* < 0.001). Given that all these factors significantly influenced serum 25(OH)D in the linear regression model, it was unsurprising that vitamin D status of the women was significantly different between the two recruitment sites. However, after adjusting for maternal age, BMI, SEI, smoking status and alcohol consumption at 15 ± 1 weeks’ gestation, ethnicity, recreational walking and season, the women recruited in Adelaide still had significantly lower serum 25(OH)D when compared with those in Auckland (Adelaide: 58.4 ± 50.3 vs. Auckland: 70.2 ± 54.5 nmol/L, P < 0.001) indicating the influence of other confounders not measured as part of the study on vitamin D status.

**Table 1:** Participant characteristics and comparison of characteristics between women recruited at the Adelaide and Auckland SCOPE centres

	All women (n = 2800)	Adelaide (n = 1156)	Auckland (n = 1644)	*P* value*
Age *yrs*, mean (SD)	28 (6)	23.73 (5.11)	30.44 (4.82)	<0.0001
BMI *kg/m*^*2*^, mean (SD)	25.8 (5.44)	27.04 (6.56)	24.88 (4.26)	<0.0001
Ethnicity				<0.0001
Caucasian	2449 (87)	1060 (92)	1389 (84)	
Non Caucasian	351 (13)	96 (8)	255 (16)	
Smoking status, n (%)				<0.0001
No	2152 (77)	704 (61)	1448 (88)	
Quit during pregnancy	306 (11)	175 (15)	131 (8)	
Smoking	342 (12)	277 (24)	65 (4)	
Alcohol Consumption, n (%)				<0.0001
No	1480 (53)	708 (61)	772 (47)	
Stopped during pregnancy	1185 (42)	397 (34)	788 (48)	
Consuming alcohol	135 (5)	51 (4)	84 (5)	
Fruit Intake, n (%)				<0.0001
≥1x per day	2032 (73)	586 (51)	1446 (88)	
3-6x per week	405 (14)	272 (24)	133 (8)	
1-2x per week	223 (8)	181 (16)	42 (3)	
1-3x per month or less	140 (5)	117 (10)	23 (1)	
Recreational Walking				<0.0001
Never	428 (15)	265 (23)	163 (10)	
1-3 times/week	1773 (63)	668 (58)	1105 (68)	
≥4 times/week	590 (22)	221 (19)	369 (23)	
Time watching TV				<0.0001
<5 hours per day	2404 (86)	901 (78)	1503 (92)	
≥5 hours per day	387 (14)	253 (22)	134 (8)	
Season serum was sampled				0.3213
Summer	636 (23)	278 (24)	358 (22)	
Autumn	705 (25)	273 (24)	432 (26)	
Winter	727 (26)	300 (26)	427 (26)	
Spring	732 (26)	305 (26)	427 (26)	
Serum 25(OH)D *nmol/L*, mean (SD)	68.09 (27.14)	60.06 (23.68)	73.74 (27.99)	<0.0001
Vitamin D Status				<0.0001
<25 nmol/L	99 (4)	48 (4)	51 (3)	
25-50 nmol/L	673 (24)	375 (32)	298 (18)	
50-75 nmol/L	928 (33)	422 (37)	506 (31)	
>75 nmol/L	1098 (39)	311 (27)	787 (48)	

*P values for continuous variables were determined using a Welch’s t-test and categorical variables a Fisher’s exact test comparing Adelaide and Auckland women

### Standardising serum 25(OH)D based on seasonal variation

**Fig. 1:**
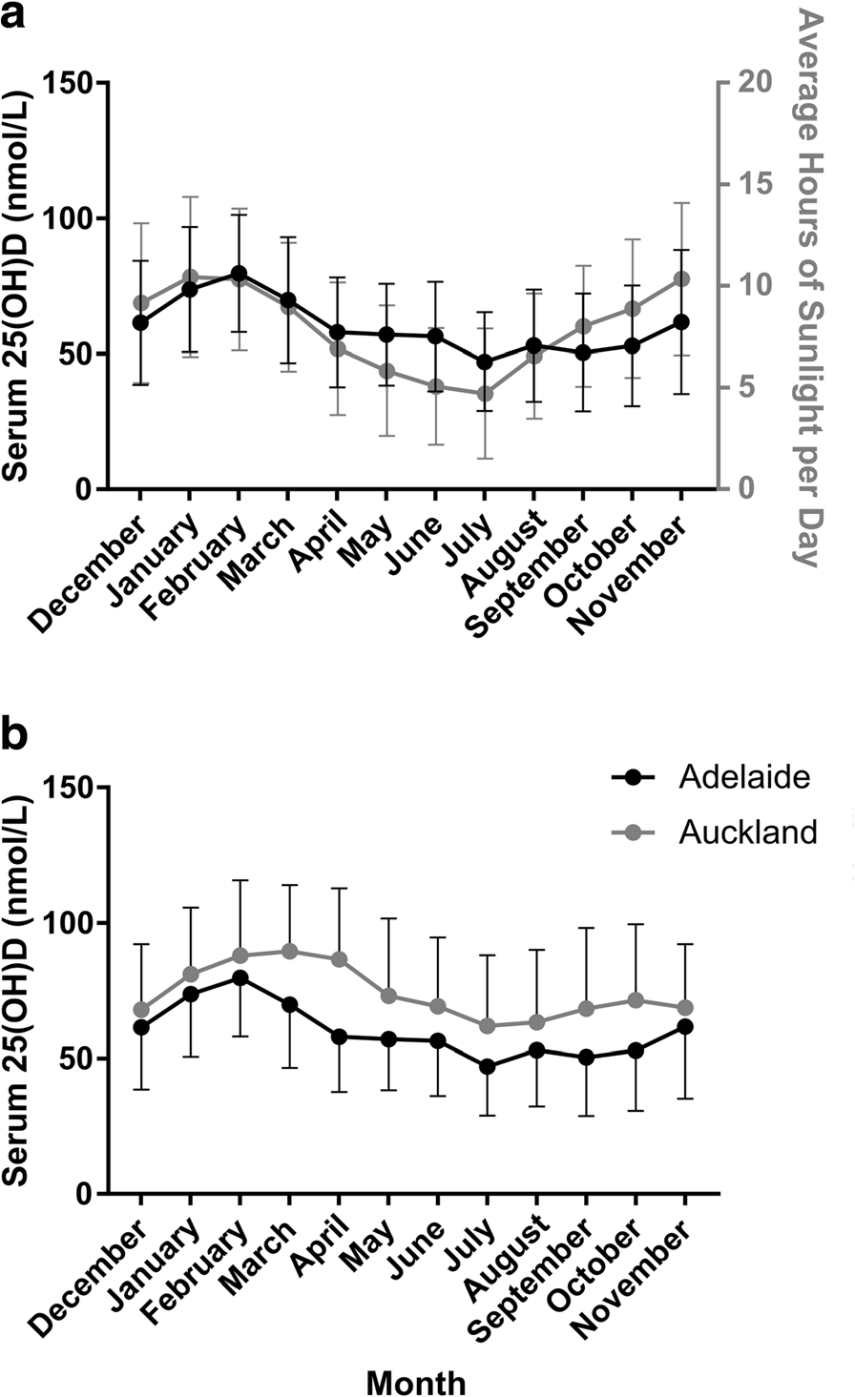
Seasonal variation in serum 25(OH)D. **a**. Comparison of serum 25(OH)D levels based on month of sampling in the women recruited in Adelaide (black line & left axis) and average hours of sunlight per day in Adelaide (grey line & right axis). Seasonal variation in vitamin D followed a similar pattern to hours of sunlight although was slightly shifted. **b**. Seasonal variation of serum 25(OH)D based on month of sampling between women recruited in Adelaide compared to Auckland. Data are mean ± SD

As expected, there was a seasonal influence on serum 25(OH)D and thus, “standardised” vitamin D concentrations were calculated to account for the month of serum collection. Average hours of sunlight per day in Adelaide from September 2005 to September 2008 were obtained from the Australian Government Bureau of Meteorology and compared against serum vitamin D measured in the women recruited in Adelaide (Fig. 1a; black line/left axis serum 25(OH)D and grey line/right axis average hours of sunlight). The means for serum 25(OH)D by month of sampling were also separated for women recruited in Adelaide and women recruited in Auckland (Fig. 1b).

### Serum 25(OH)D and pregnancy outcome

Of the 2800 women present in this study, 1217 (43%) women went on to develop a pregnancy complication and included 5.8% (*n* = 161) who developed PE, 7.6% (*n* = 213) who developed GH, 3.3% (*n* = 92) who developed GDM, 5% (*n* = 139) who spontaneously delivered preterm and 10.6% (*n* = 298) who delivered an SGA infant.

The association between “standardised” 25(OH)D with pregnancy complications is presented in Table 2. Using moderate-high as a reference (standardised vitamin D 63–81 nmol/L), there was no appreciable effect of having a low, low-moderate or high vitamin D with the risk of developing any pregnancy complication after adjusting for confounders. However, when each pregnancy complication was analysed separately, a 53% decreased risk for GDM was observed with high vitamin D status when compared to moderate-high status (aRR: 0.47; 95% CI: 0.23, 0.96). When women were categorised based on clinical definitions of vitamin D deficiency and serum 25(OH)D between 50 and 75 nmol/L used as the reference, no significant relationship between vitamin D status and adverse pregnancy outcome was found (Table 2). Although, the point estimates indicated a marginal increased risk for developing any pregnancy complication with severe (< 25 nmol/L) vitamin D deficiency compared to those who had levels between 50 and 75 nmol/L (aRR: 1.10; 95% CI: 0.89, 1.36).

**Table 2:** Adjusted relative risks (aRR) of pregnancy complications from any complication, preeclampsia (PE), gestational hypertension (GH), gestational diabetes mellitus (GDM), spontaneous preterm birth (sPTB) and small-for-gestational age (SGA) according to vitamin D status categorised based on standardised quartiles and clinical definitions of vitamin D status

		Pregnancy Complications											
	All women	Any Complication		PE		GH		GDM		sPTB		SGA	
	*n* (%)	*n* (%)	aRR* (95% CI)	*n* (%)	aRR* (95% CI)	*n* (%)	aRR* (95% CI)	*n* (%)	aRR* (95% CI)	*n* (%)	aRR* (95% CI)	*n* (%)	aRR* (95% CI)
Standardised Quartiles**													
Low	711 (25)	328 (46)	0.95 (0.85, 1.07)	46 (6)	1.02 (0.66, 1.57))	57 (8)	0.97 (0.67, 1.42)	28 (4)	0.79 (0.47, 1.30)	36 (5)	0.83 (0.54, 1.29)	84 (12)	1.02 (0.75, 1.39)
Low-Moderate	755 (27)	318 (42)	1.00 (0.90, 1.12)	42 (6)	1.07 (0.69, 1.64)	65 (9)	1.06 (0.74, 1.52)	25 (3)	0.67 (0.39, 1.16)	33 (4)	0.87 (0.56, 1.35	72 (10)	0.98 (0.73, 1.32)
Moderate-High	702 (25)	309 (44)	1.0†	37 (5)	1.0†	50 (7)	1.0†	28 (4)	1.0†	39 (6)	1.0†	81 (12)	1.0†
High	630 (23)	258 (41)	0.93 (0.82, 1.06)†	35 (6)	1.24 (0.78, 1.96)	40 (6)	0.87 (0.58, 1.30)	10 (2)	**0.47** **(0.23, 0.96)**	30 (5)	0.77 (0.48, 1.25)	60 (10)	0.80 (0.57, 1.11)
Vitamin D Status													
<25 nmol/L	99 (4)	51 (52)	1.10 (0.89, 1.36)	8 (8)	1.44 (0.68, 3.03))	6 (6)	0.70 (0.32, 1.52)	5 (5)	0.98 (0.38, 2.52)	7 (7)	1.44 (0.68, 3.04)	13 (13)	1.15 (0.66, 2.01)
25-50 nmol/L	673 (24)	301 (45)	0.93 (0.83, 1.03)	45 (7)	1.18 (0.80, 1.75)	59 (9)	0.97 (0.71, 1.34)	26 (4)	0.89 (0.54, 1.47)	29 (4)	0.75 (0.48, 1.16)	74 (11)	0.93 (0.69, 1.24)
>50-75 nmol/L	928 (33)	428 (46)	1.0†	48 (5)	1.0†	80 (9)	1.0†	35 (4)	1.0†	52 (6)	1.0†	107 (12)	1.0†
>75 nmol/L	1098 (39)	435 (40)	0.95 (0.86, 1.04)	60 (5)	1.28 (0.87, 1.87)	67 (6)	0.82 (0.59, 1.12)†	26 (2)	0.78 (0.48, 1.28)	51 (5)	0.86 (0.59, 1.26)	104 (9)	0.86 (0.67, 1.12)

*Relative risks compared to all women were adjusted for age, maternal body mass index, ethnicity (non Caucasian vs. Caucasian), smoking status at 15 ± 1 weeks’ gestation (no vs. yes), alcohol consumption at 15 ± 1 weeks’ (no vs yes), recreational walking (1-3×/week and ≥ 4×/week vs. never) and recruitment site (Auckland vs. Adelaide)

**Serum 25(OH)D was standardised for month that serum was sampled based as previously described [34]

†Reference category

### Serum 25(OH)D, pregnancy outcome and fetal sex

As there is evidence to suggest that vitamin D metabolism within the placenta may differ with respect to fetal sex [35] thus, we assessed the effect of “standardised” vitamin D status on pregnancy outcome stratified by fetal sex (Table 3). Although not statistically significant, point estimates for women carrying a male fetus indicated decreased risk of having any pregnancy complication with high serum 25(OH)D compared to moderately-high (aRR: 0.86; 9%% CI: 0.71, 1.04). This was largely driven by a 55% decreased risk of GDM in women with a male fetus with high standardised serum 25(OH)D when compared to those carrying a male fetus with moderate-high serum 25(OH)D (aRR: 0.45; 95% CI: 0.15, 1.38). Conversely, if a woman was carrying a female fetus, there was generally no effect of having low, low-moderate or high standardised serum 25(OH)D on developing any pregnancy complication compared to moderate-high levels (Table 3). Although, a 56% decreased risk for GDM was observed in those with low-moderate levels of standardised vitamin D when compared to moderate-high levels for women carrying a female fetus (aRR: 0.44; 95% CI: 0.20, 0.97). Similar results were observed when vitamin D status was based on clinical definitions of deficiency (Additional file 1: Table S1).

**Table 3:** Adjusted relative risks (aRR) of pregnancy complications from any complication, preeclampsia (PE), gestational hypertension (GH), gestational diabetes mellitus (GDM), spontaeous preterm birth (sPTB) and small-for-gestational age (SGA) according to vitamin D status and stratified by fetal sex

		Pregnancy Complications											
	All women	Any Complication		PE			GH		GDM		sPTB		SGA
	*n* (%)	*n* (%)	aRR* (95% CI)	*n* (%)	aRR* (95% CI)	*n* (%)	aRR* (95% CI)	*n* (%)	aRR* (95% CI)	*n* (%)	aRR* (95% CI)	*n* (%)	aRR* (95% CI)
Standardised Quartiles**													
Males													
Low	367 (26)	173 (47)	1.00 (0.85, 1.18)	24 (7)	1.24 (0.65, 2.34)	31 (8)	1.11 (0.64, 1.94)	18 (5)	1.25 (0.58, 2.68)	22 (6)	1.09 (0.60, 1.98)	43 (12)	1.14 (0.73, 1.79)
Low-Moderate	364 (26)	163 (45)	1.01 (0.86, 1.19))	20 (5)	1.11 (0.58, 2.12)	32 (9)	1.25 (0.73, 2.13)	13 (4)	1.06 (0.48, 2.38)	18 (5)	0.93 (0.49, 1.76)	45 (12)	1.23 (0.80, 1.88)
Moderate-High	359 (25)	152 (42)	1.0†	16 (4)	1.0†	23 (6)	1.0†	11 (3)	1.0†	18 (5)	1.0†	35 (10)	1.0†
High	325 (23)	111 (34)	0.86 (0.71, 1.04)	15 (5)	1.18 (0.59, 2.36)	17 (5)	0.90 (0.50, 1.62)	4 (1)	0.45 (0.15, 1.38)	15 (5)	0.97 (0.50, 1.88)	26 (8)	0.77 (0.47, 1.26)
Females													
Low	380 (28)	169 (44)	0.91 (0.77, 1.06)	23 (6)	0.85 (0.47, 1.55)	30 (8)	0.87 (0.52, 1.45)	13 (3)	0.51 (0.25, 1.04)	14 (4)	0.61 (0.31, 1.18)	45 (12)	0.88 (0.58, 1.35)
Low-Moderate	353 (26)	168 (48)	0.99 (0.85, 1.15)	24 (7)	1.03 (0.57, 1.85)	31 (9)	0.92 (0.57, 1.50)	9 (3)	**0.44** **(0.20, 0.97)**	18 (5)	0.79 (0.43, 1.45)	33 (9)	0.74 (0.48, 1.13)
Moderate-High	334 (24)	149 (45)	1.0†	19 (6)	1.0†	28 (8)	1.0†	17 (5)	1.0†	21 (6)	1.0†	31 (9)	1.0†
High	314 (23)	128 (41)	1.00 (0.84, 1.19)	19 (6)	1.29 (0.70, 2.35)	20 (6)	0.85 (0.49, 1.46)	6 (2)	0.48 (0.19, 1.23)	12 (4)	0.60 (0.30, 1.21)	29 (9)	0.82 (0.53, 1.28)

*Relative risks compared to all women were adjusted for age, maternal body mass index, ethnicity (non Caucasian vs. Caucasian), smoking status at 15 ± 1 weeks’ gestation (no vs. yes), alcohol consumption at 15 ± 1 weeks’ (no vs yes), recreational walking (1-3×/week and ≥ 4×/week vs. never) and recruitment site (Auckland vs. Adelaide)

**Serum 25(OH)D was standardised for month that serum was sampled based as previously described [34]

†Reference category

## Discussion

This study adds to the current body of literature on vitamin D status in a population of pregnant Australian and New Zealand women and provides insight into normal circulating levels of 25(OH)D in early pregnancy. It is the largest prospective cohort study to assess vitamin D status within the international SCOPE cohort, comparing and combining two distinct populations of pregnant women living at similar latitude and provides a greater understanding of vitamin D deficiency and the risk of adverse pregnancy outcomes. We found, despite being at similar latitudes, circulating 25(OH)D was different between women recruited in Adelaide compared to women recruited in Auckland. This was independent of diet and lifestyles factors including BMI and SEI and highlights the difficulty in understanding the role of vitamin D in pregnancy in human cohort studies. However, there was a protective association of having high vitamin D at 15 ± 1 weeks’ gestation and GDM once standardised based on month serum was sampled. Furthermore, there may be possible fetal sex specific differences in vitamin D status worth considering in future studies.

Despite the similar latitudes of Adelaide and Auckland (Adelaide: 34.93°S and Auckland 36.85°S), serum 25(OH)D was lower in the women recruited in Adelaide. Given the significant number of characteristic and lifestyle differences between the two populations, this is not overly surprising. Previous studies have shown a positive association between socioeconomic status and vitamin D in both pregnant and non-pregnant women [36, 37]. The lower SEI of the Adelaide women could therefore make them more susceptible to lower circulating 25(OH)D because of factors relating to disadvantage. Furthermore, increased BMI is known to be associated with reduced serum 25(OH)D [38–40] as adipose tissue is thought to sequester 25(OH)D [41]. However, after adjusting for factors shown to be associated with vitamin D status including BMI and recreational walking, serum 25(OH)D remained significantly lower in the women recruited in Adelaide suggesting the influence of other factors not measured as part of SCOPE for example, hours spent outside in the sun. As indicated by the seasonal variation in serum 25(OH)D, amongst women recruited in Adelaide, vitamin D status declined significantly from February to April whilst in the women recruited in Auckland, serum 25(OH)D in March and April remained elevated before declining.

Vitamin D deficiency has previously been associated with insulin resistance and type 2 diabetes given its role in supporting insulin secretion and pancreatic β-cell function [42]. Furthermore, inverse relationships between serum 25(OH)D and both fasting glucose and fasting insulin have been shown during pregnancy indicating poorer glycaemic control [19, 43]. Therefore, the protective role of high 25(OH)D 15 ± 1 weeks’ gestation against GDM, as seen with a decreased risk of GDM in women within the ‘high’ quartile of standardised vitamin D compared to moderate-high, is consistent with knowledge about vitamin D and diabetes. This is also consistent with another study that observed an increased risk of GDM with vitamin D deficiency in early pregnancy (aOR: 3.40; 95% CI: 2.03–4.98 [17]) and offers potential physiological connections between vitamin D status and the progression of insulin resistance in pregnant women. Furthermore, studies have shown that vitamin D supports early placental development [44, 45] in which, abnormal placentation can be a characteristic of a number of pregnancy complications including GDM, PE, sPTB and SGA [46].

The placenta expresses all the necessary components to convert 25(OH)D to the active form and thus utilise active vitamin D either locally or in a paracrine manner [45, 47]. Vitamin D metabolism in the placenta has been shown to be influenced by testosterone production and thus varies by fetal sex [35]. Furthermore, sex specific differences in pregnancy outcome have also been reported whereby the risk of sPTB, PE and GDM are all higher in pregnancies with a male fetus [48–50]. In this study, we observed marginal sex-specific differences between early pregnancy vitamin D status and pregnancy outcome where by high vitamin D status and carrying a male fetus was moderately associated with decreasing the risk of having any pregnancy complication. Conversely, high vitamin D status and carrying a female fetus was not associated with changing the risk of having any pregnancy complication. Indeed, for the risk of GDM an opposite effect of vitamin D status in early pregnancy was observed depending on fetal sex. This is similar to that which has previously been shown in the relationship between vitamin D status at ≤26 weeks’ gestation and placental pathology in pregnancy [51] suggesting that male and female fetuses respond differently to maternal vitamin D status.

Lack of statistically significant associations with other pregnancy complications may reflect the fact that this was a largely vitamin D-replete population (> 72% with serum 25(OH)D > 50 nmol/L) likely due to their residence latitude as low serum 25(OH)D was found in the SCOPE Ireland cohort [52]. Indeed many of the studies that have assessed the association between vitamin D status and adverse pregnancy outcome that have reported statistically significant differences have been in populations with higher rates of vitamin D deficiency [2, 4]. High dose (> 2000 IU) vitamin D supplementation is associated with decreasing risk of pregnancy complications [23–25]. However, routine vitamin D supplementation did not occur during the time period the women in the current study were recruited. Furthermore, multivitamin supplements available during the study period contained very little (maximum 50 IU), if any, vitamin D and thus are an unlikely source of variation within the population. Inconsistencies in the literature may also reflect other causative factors in which vitamin D is a mediator. For example, active vitamin D (1,25-dihydroxyvitamin D_3_) is the principal hormone that regulates calcium absorption within the intestine [53] and is integral to maintaining calcium homeostasis. During gestation, fetal demand for calcium increases and it is imperative that maternal vitamin D status remain adequate to support increased calcium absorption from the gut [54]. Therefore, vitamin D status may be important in populations where dietary calcium intake is low which is unlikely in the population of women studied here.

## Conclusions

In conclusion, once standardised against month of sampling, we demonstrate a protective effect of high vitamin D with GDM. However, differences in vitamin D status between the women recruited in Adelaide and those recruited in Auckland reflect obvious difficulties in studying how vitamin D may support healthy pregnancies. A possible connection between fetal sex, vitamin D status and pregnancy complications reveals further questions and encourages continual research and discussion into the role of vitamin D in pregnancy, particularly in vitamin D replete populations.

## Additional file

Additional file 1: **Table S1.** Adjusted relative risks (aRR) of pregnancy complications from any complication, preeclampsia (PE), gestational hypertension (GH), gestational diabetes mellitus (GDM), spontaeous preterm birth (sPTB) and small-for-gestational age (SGA) according to vitamin D status and stratified by fetal sex. (DOCX 15 kb)

## Abbreviations

25(OH)D: 25-hydroxy vitamin D
aRR: adjusted risk ratio
BMI: body mass index
CI: confidence interval
GDM: gestational diabetes mellitus
GH: gestational hypertension
PE: preeclampsia
SCOPE: SCreening fOr Pregnancy Endpoints
SEI: socioeconomic index
SGA: small for gestational age
sPTB: spontaneous preterm birth
