# Supplementary material for: Vitamin D levels in an Australian and New Zealand cohort and the association with pregnancy outcome
Source: BMC Pregnancy Childbirth. 2018 Jun 20;18:251. doi: 10.1186/s12884-018-1887-x (PMC6011374; doi:10.1186/s12884-018-1887-x)
Supplement: Supplementary file 1 — Table S1. Adjusted relative risks (aRR) of pregnancy complications from any complication, preeclampsia (PE), gestational hypertension (GH), gestational diabetes mellitus (GDM), spontaeous preterm birth (sPTB) and small-for-gestational age (SGA) according to vitamin D status and stratified by fetal sex. (DOCX 15 kb) [file 12884_2018_1887_MOESM1_ESM.docx]

**Supplemental Table 1** Adjusted relative risks (aRR) of pregnancy complications from any complication, preeclampsia (PE), gestational hypertension (GH), gestational diabetes mellitus (GDM), spontaeous preterm birth (sPTB) and small-for-gestational age (SGA) according to vitamin D status and stratified by fetal sex

|  |  | Pregnancy Complications | | | | | | | | | | | |
| --- | --- | --- | --- | --- | --- | --- | --- | --- | --- | --- | --- | --- | --- |
|  | All women | Any Complication | | PE | |  | GH |  | GDM |  | sPTB |  | SGA |
|  | *n* (%) | *n* (%) | aRR*  (95% CI) | *n* (%) | aRR*  (95% CI) | *n* (%) | aRR*  (95% CI) | *n* (%) | aRR*  (95% CI) | *n* (%) | aRR*  (95% CI) | *n* (%) | aRR*  (95% CI) |
| Vitamin D Status | | | |  |  |  |  |  |  |  |  |  |  |
| Males |  |  |  |  |  |  |  |  |  |  |  |  |  |
| <25 nmol/L |  |  | 1.18  (0.87, 1.60) |  | 0.88  (0.20, 3.82) |  | 0.48  (0.11, 2.02) |  | 1.29  (0.37, 4.50) |  | 1.78  (0.69, 4.57) |  | 0.79  (0.29, 2.12) |
| 25-50 nmol/L |  |  | 1.01  (0.87, 1.17) |  | 1.32  (0.75, 2.33) |  | 0.98  (0.62, 1.53) |  | 1.05  (0.55, 2.01) |  | 1.12  (0.63, 2.00) |  | 0.99  (0.67, 1.46) |
| >50-75 nmol/L |  |  | 1.0† |  | 1.0† |  | 1.0† |  | 1.0† |  | 1.0† |  | 1.0† |
| >75 nmol/L |  |  | 0.95  (0.82, 1.10) |  | 1.20  (0.69, 2.09) |  | 0.69  (0.44, 1.10) |  | **0.47**  **(0.22, 1.00)** |  | 1.04  (0.61, 1.79) |  | 0.75  (0.52, 1.08) |
| Females |  |  |  |  |  |  |  |  |  |  |  |  |  |
| <25 nmol/L |  |  | 1.04  (0.78, 1.39) |  | 1.81  (0.75, 4.34) |  | 0.91  (0.37, 2.26) |  | 0.73  (0.17, 3.10) |  | 1.25  (0.38, 4.03) |  | 1.46  (0.75, 2.83) |
| 25-50 nmol/L |  |  | 0.85  (0.73, 1.01) |  | 1.08  (0.62, 1.88) |  | 1.00  (0.63, 1.60) |  | 0.70  (0.32, 1.52) |  | **0.41**  **(0.19, 0.88)** |  | 0.89  (0.58, 1.37) |
| >50-75 nmol/L |  |  | 1.0† |  | 1.0† |  | 1.0† |  | 1.0† |  | 1.0† |  | 1.0† |
| >75 nmol/L |  |  | 0.94  (0.82, 1.08) |  | 1.36  (0.80, 2.31) |  | 0.95  (0.61, 1.48) |  | 1.19  (0.60, 2.34) |  | 0.69  (0.40, 1.19) |  | 1.06  (0.74, 1.51) |
| *Relative risks compared to all women were adjusted for age, maternal body mass index, ethnicity (non Caucasian vs. Caucasian), smoking status at 15±1 weeks' gestation (no vs. yes), alcohol consumption at 15±1 weeks' (no vs yes), recreational walking (1-3x/week and ≥4x/week vs. never) and recruitment site (Auckland vs. Adelaide).  †Reference category | | | | | | | | | | | | | |
